# Nurses’ perceived role in healthcare transition of adolescents with intellectual disabilities

**DOI:** 10.4102/ajod.v9i0.674

**Published:** 2020-12-14

**Authors:** Grace R. Malapela, Gloria Thupayagale-Tshweneagae, Olabisi F. Ibitoye

**Affiliations:** 1 Department of Health Studies, College of Human Sciences, University of South Africa, Pretoria, South Africa

**Keywords:** adolescents, adulthood, intellectual disabilities, nurses, role, transition

## Abstract

**Background:**

Nurses’ role in the transition of adolescents living with intellectual disabilities (IDs) has always been neglected. The primary role of nurses is to promote health, provide nursing care, alleviate suffering and rehabilitate. Improving the quality of life for individuals with ID when they undergo the healthcare transition process from adolescence to adulthood was previously not considered a priority.

**Objectives:**

To investigate the nurses’ perceived role in healthcare transition of adolescents with ID into adulthood.

**Method:**

A qualitative descriptive design using semi-structured interviews with 18 nurse participants was used. The sample included 25 nurses working in care and rehabilitation centres and non-governmental organisations in Tshwane district, Gauteng province, South Africa.

**Results:**

Three main themes that emerged from the analysis were support systems, advocacy and health promotion.

**Conclusion:**

The findings of this study clearly show the need for a strong healthcare support system to facilitate a successful transition process of adolescents with ID. The study findings support the view that nurses can play a key role in assisting individuals with ID and their families in dealing with the challenges of healthcare transitioning into adulthood.

## Introduction

Nurses’ role in the transition of adolescents living with intellectual disabilities (IDs) has always been neglected (Betz 2003). The primary role of nurses is to promote health, provide nursing care, alleviate suffering and rehabilitate. Nurses should provide supervision, direction and support in the management of adolescents with ID, with the aim of optimising the life and health of individuals and their families.

Improving the quality of life for individuals with ID when they undergo the healthcare transition process from adolescence to adulthood was previously not provided or considered a priority.

Transition into adulthood is universally challenging for most adolescents. Adolescents with ID face more difficulties than those without ID owing to their cognitive and behavioural limitations (Ally et al. 2018). Transition is multifaceted.

Transition includes progression from school to commencement of work life, social and community participation and independent living from the family (Cobb & Alwell 2009). An individual with disability also needs healthcare transition services from paediatric to adult healthcare settings (Betz, Nehring & Lobo 2015). The purpose of the current study is to provide insights into nurses’ perceptions of their role in healthcare transition of adolescents with ID.

Several studies have documented poor preparation and coordination of services for young adults with special need for the transition process to adulthood (Doug et al. 2011; Rutishauser, Akré & Surìs 2011). This lack of proper planning and support hinders them from achieving an expected level of independence (Lotstein et al. 2005). Successful transition for people with ID requires partnership and collaborative efforts of a wide range of professionals, agencies, centres or services. Previous studies on transition for people with ID mainly focused on independent living, employment, interpersonal and intimate relationships, whilst only few studies were conducted on healthcare transition (Betz et al. 2015).

According to Blum et al. (1993), healthcare transition is the:

[*P*]urposeful, planned movement of adolescents and young adults with chronic physical and medical conditions from child-centred to adult-oriented health care systems that is uninterrupted, coordinated, developmentally appropriate, psychosocially sound, and comprehensive. (p. 570)

It facilitates the transfer of care from paediatric to adult healthcare and supports the acquisition of developmental needs.

The American Academy of Pediatrics, American Academy of Family Physicians and American Society of Internal Medicine define healthcare transition as (American Academy of Pediatrics, American Academy of Family Physicians & American College of Physicians-American Society of Internal Medicine 2002):

[*A*] dynamic, lifelong process that seeks to meet their individual needs as they move from childhood to adulthood. The goal is to maximise lifelong functioning and potential through the provision of high quality, developmentally appropriate health care services that continue uninterrupted as the individual moves from adolescence to adulthood. It is client-centred, and its cornerstones are flexibility, responsiveness, continuity, comprehensiveness, and coordination. (p. 1304)

Healthcare transition planning is the responsibility of the care providers such as the primary care physicians, nurse practitioners and physician assistants as well as medical subspecialists, but performing these roles can also be a complex process as the activities involved during this period go far beyond the routine care for simple chronic disease (Okumura, Saunders & Rehm 2015). There is evidence that nurses were not actively involved in the process (Betz et al. 2015). This lack of involvement and participation has been attributed to the poor definition of the scope of nursing practice roles and responsibilities in healthcare transition. Al-Yateem and Docherty (2015) asserted that transition is regarded as a concept of significance to nursing and healthcare professionals. Nurses should be active participants in all stages of transition for adolescents with ID. However, studies have also suggested unwillingness of some adult-focused healthcare providers in caring for children with chronic disability conditions, whilst a lack of resources for the care providers to prepare youth for transition was also reported (Okumura et al. 2008; Sharma et al. 2014). Evidence-based approaches to provide healthcare transition services remain obscure for nurses, especially in South Africa. This necessitates the need to investigate the nurses’ perceived role in the transition of adolescents with ID.

## Research question

This study aims at answering the following question: what are the nurses’ perceived roles in healthcare transition of adolescents with ID?

## Research design and methods

Sandelowski’s (2010) descriptive qualitative design was adopted for this study. The researchers’ aim was to explore what the nurses perceive as their role in the healthcare transition of adolescents with ID.

### Participants

The study population included 18 nurses working in non-governmental organisations (NGOs) and care and rehabilitation centre in Tshwane district, Gauteng province, South Africa. All the nurses were registered with the South African Nursing Council (SANC) which is a statutory body. Eight participants had a Diploma in Nursing (General, Psychiatric Community) and Midwifery (R.425), five participants had a Diploma in Psychiatric Nursing (R 880) and five participants had a Diploma in General Nursing (R.683). The sample was selected through purposive sampling method. Nurses above 21 years of age who were directly involved in the care, treatment and rehabilitation irrespective of race and gender were included in this study. Nurses younger than 21 years were excluded from the study.

### Data collection

A total of 18 individual semi-structured interviews were conducted between December 2016 and September 2017. Participants signed a consent form before the commencement of interviews. The majority of interviews were conducted at the participants’ places of work, except for three participants who opted to be interviewed at a venue agreed upon between the first author and them. The interview guide was developed by the authors and was used to solicit information on the participants’ perceived role in healthcare transition of adolescents with ID. Each interview started with a broad statement: ‘tell me your role as a nurse in the healthcare transition of adolescents living with intellectual disabilities into adulthood’. This statement was followed by probes in the form of questions or statements depending on the answer. Such probes include the following: ‘is this how you view your role?’ and ‘what do you think should be done to ensure clarity of your role in the transition of adolescents with intellectual disability?’

### Procedure

Recruitment of participants took 3 months because of delays in response by other institutions. Permission letter, ethical clearance certificate, information leaflet and consent forms were sent via email to the institutions for approval to conduct the study. After approval and consent were granted, interviews were conducted at the convenient time during breaks and rest days in order to gain cooperation from the participants. In each of the three centres, an office with enough lighting and space was allocated for the study. The office was usually located at one corner of the centre away from movements to avoid distractions. The interviews lasted for 30–45 min depending on the participants’ response. Data saturation was reached on the 11th participant. However, the interviews continued until Participant 18.

### Data analysis

Sandelowski’s (2010) approach to data analysis was used in this study. In accordance with the approach, data analysis started at the same time with data collection. The transcripts were read several times by the first and second authors to get an in-depth understanding of the participants’ views. Interviews were systematically coded using Sandelowski’s (2010) approach in order to explore emergent themes on the perceptions of nurses regarding their role in the transition of adolescents with ID. Five of the transcripts were given to an independent coder for accuracy checking, and there were only minor differences in the wording. All the differences were discussed and consensus was reached. Three themes emerged from the analysis.

### Ethical consideration

The Higher Degrees Committee at the Department of Health Studies, University of South Africa (UNISA), approved the study protocol (Reference number: HSHDC/540/2016). The Gauteng Department of Health also granted permission (Reference number: GP_2017RP23_259). Furthermore, permission was also obtained from all the three institutions where the study was conducted. More importantly, participants signed an informed consent form before data collection.

## Findings

### Demographic profile

Participants were registered professional nurses, with their ages ranging from 29 to 59 years. Most of them were female participants and married (*n* = 13) and only one was a male participant with children. Fourteen participants were working in government care and rehabilitation centre, and only four out of 18 participants were working in NGOs.

After rigorous analysis of the interview transcripts, three major themes emerged, namely, support systems, advocacy role and health promotion.

### Support systems

The first major theme from this study was the role of nurses as a ‘support system’ for individuals with ID and their families. Nurses are an integral part of the health team and the first contact point in most care rendering services. In addition, nurses need to play a major role in planning for transition process and ensure that they provide support to the families, relatives and individuals with ID during this journey. The support services identified here include physical, information, social and psychological support.

The understanding of this important role was apparent in the following extracts:

‘As nurses we should ensure that we are there for the patients to ensure better health and positive outcomes for all irrespective of colour, race and disability.’ (Female, 34 years old)

‘In most cases, families lack information on how to access some of the service systems and other health care systems where they can be helped. Nurses need to know the available facilities and services in the communities so that families, relatives and individuals with intellectual disabilities might benefit. Services like social, education, transport, psychological, occupational, spiritual, non-governmental organisations and private enterprise might assist in reducing some of the services.’ (Female, 50 years old)

‘It is the duty of the nurse to ensure that individuals with intellectual disabilities are physically, socially, psychologically and spiritually healthy to be able to comply with the transition process. This also will promote the health and well-being of the individuals and their families.’ (Female, 29 years old)

Performing the supporting role was expressed to be difficult sometimes, especially in facilities with less human resources:

‘With more workload and staff shortage, less time is spent with the individuals and families. As a result we miss a lot and as a result patients tend to regress rather than progress.’ (Male, 40 years old)

### Advocacy role

The need for nurses to step into advocacy role for the adolescents with ID was emphasised as this group has less power to fight for themselves and sometimes the family might feel overwhelmed:

‘Parents are often confused and uncertain about what the future holds for their children. … Failure of nurses to advocate for clients’ rights makes them more prone to abuse, stigma and discrimination.’ (Female, 30 years old)

‘Nurses have the role to advocate for the patients and to act in the best interest of the patients so that they achieve quality of life.’ (Male, 40 years old)

The nurses ensure that this group of people have access to other service systems with constraints as most facilities are ill-equipped to render some required services:

‘The challenge is that we are far from everything. Our institution does not have a learning centre where these individuals can learn most of the working skills and be able to earn a living and be employable.’ (Female, 45 years old)

‘What we do is just to provide basic care and ensure that their basic needs are met. No preparations towards the future. Although others are profoundly disabled, others can benefit. This will bring about positive change and outcomes into their lives. As a result, they will be able to contribute to the community and be valuable members in the community that bring about change.’ (Female, 39 years old)

### Health promotion

Nurses in the study highlighted the lack of awareness about the transition process as a barrier to proper transition.

To this end, the participants conveyed the need for an interdisciplinary approach to strengthen and promote more awareness for individual, family and community about transition process and the support services available:

‘Lack of awareness is one of the barriers to the transition process. More platforms at community level should be initiated so that communities can [*come*] up with new ways to support these individuals and their families. Transition process should be regarded as a societal issue rather than being a family or health issue.’ (Female, 42 years old)

‘These people can contribute something positive to the community. But they just have to sit at home, do nothing and expect nothing in return.’

‘Without training and counselling, transition process and its challenges will not be reduced. Parents tend to be reluctant as far as transition process is concerned. Service systems that cater for individuals with intellectual disabilities are limited. Better services are available in private sectors, but expensive. Therefore, affordability becomes a challenge.’ (Female, 39 years old)

## Discussion

This study found that nurses are the support system for both the families and individuals with ID. According to Ally et al. (2018), transition process is not easy, and hence families need to be supported through the healthcare transition of adolescents with ID to adulthood. For nurses to effectively provide support to the families and individuals with ID, they need to involve other stakeholders such as other healthcare professionals, social workers and others in both the planning and execution of the support system.

McNally and Mannan (2013) asserted that support systems make caring for individuals with ID less stressful. Lima-Rodriguez et al. (2018) added that supporting families would promote the best outcomes for everyone, as families spend most of their time with their children. Lindgren, Soderberg and Skar (2014) suggested that transition planning can be achieved only through support.

In accordance with Article 25 of the United Nations Convention of Rights of Persons with Disabilities cited in Lennox, McPherson and Van Dooren (2015), individuals with ID have the right to receive the highest standard of living without being discriminated. This calls upon nurses and other services to work together in ensuring that rights of individuals with ID are not compromised. In South Africa, *the Mental Health Act* No. 17 of 2002, Chapter III (Section 8), outlines that special precautions should be considered to ensure that persons designated in the care, treatment and rehabilitation services act in the best interest of the users with mental health problems. Furthermore, the policy guidelines of child and adolescent mental health in South African National Department of Health (NDoH) (2003) reported that people should speak up, limit social discrimination and create opportunities for employment, recreation, schooling and reduction of socio-economic inequalities.

The nurses also perceived their role as that of advocacy. Transition advocacy has been recognised as an essential tool for transitioning youth, families and providers’ need to ensure care. Through this process, the care provider can work with the youth and parents to obtain needed resources for the transition and eventual transfer process. Nurses as care providers are not expected to focus on chronic disease management alone, and provision of information regarding available resources is not sufficient to ensure successful transition (Okumura et al. 2015). Furthermore, nurses should assist with navigating and managing resources. In addition, Clarke, Camilleri and Goding (2015) stated that advocacy is central to the experience of self-esteem, self-development and empowerment.

The other finding of the study was on health promotion as one of the nurses’ perceived roles in the transition of adolescents with ID. According to McNally and Mannan (2013), the demand of caring for individuals with ID is high and challenging. The nurses’ role in the promotion of health should include communicating with other stakeholders on the availability of services aimed at promoting the health of individuals with disabilities and their families. Such services may include counselling services, rehabilitation services and social services for the provision of financial assistance and others. Zhou et al. (2016) concluded that structured multidisciplinary transition programmes are necessary for the promotion of health. Therefore, healthcare services should ensure that relevant services are in place and accessible.

## Clinical and research implications

Evidence from this study indicates a need for a multi-sectoral approach where all stakeholders could be involved in the transition planning of adolescents with ID. The roles played by all stakeholders will assist individuals with ID to have a successful transition into adulthood and to be situated in their communities.

Further research on the role of nurses with the transition process of adolescents with ID is needed. Additional research and information from other healthcare providers and practitioners to explore their specific roles with regard to the transition of adolescents with ID into adulthood is essential. Personalised transition needs should also consider the nature, extent and degree of the intellectual disability.

## Limitations

This study was limited to a few care and rehabilitation centres and NGOs in the Gauteng province. Other healthcare providers and mental healthcare practitioners did not take part in this study. Therefore, the generalisation of the study findings is limited.

## Conclusion

The nurses in this study perceived their roles as that of support, advocacy and health promotion. The study showed that the transition of adolescents with ID into adulthood would need a combined effort from all stakeholders to make it a success.
